# The immune-suppressive landscape in lepromatous leprosy revealed by single-cell RNA sequencing

**DOI:** 10.1038/s41421-021-00353-3

**Published:** 2022-01-11

**Authors:** Zihao Mi, Zhenzhen Wang, Xiaotong Xue, Tingting Liu, Chuan Wang, Lele Sun, Gongqi Yu, Yuan Zhang, Peidian Shi, Yonghu Sun, Yongliang Yang, Shanshan Ma, Zhe Wang, Yueqian Yu, Jianjun Liu, Hong Liu, Furen Zhang

**Affiliations:** 1 Shandong Provincial Hospital for Skin Diseases & Shandong Provincial Institute of Dermatology and Venereology, Shandong First Medical University & Shandong Academy of Medical Sciences, Jinan, Shandong China; 2 Human Genetics, Genome Institute of Singapore, Singapore, Singapore; 3 Shandong Provincial Hospital Affiliated to Shandong First Medical University, Jinan, Shandong China

**Keywords:** Gene expression profiling, Immunology

## Abstract

Lepromatous leprosy (L-LEP), caused by the massive proliferation of *Mycobacterium leprae* primarily in macrophages, is an ideal disease model for investigating the molecular mechanism of intracellular bacteria evading or modulating host immune response. Here, we performed single-cell RNA sequencing of both skin biopsies and peripheral blood mononuclear cells (PBMCs) of L-LEP patients and healthy controls. In L-LEP lesions, we revealed remarkable upregulation of *APOE* expression that showed a negative correlation with the major histocompatibility complex II gene *HLA-DQB2* and *MIF*, which encodes a pro-inflammatory and anti-microbial cytokine, in the subset of macrophages exhibiting a high expression level of *LIPA*. The exhaustion of CD8^+^ T cells featured by the high expression of *TIGIT* and *LAG3* in L-LEP lesions was demonstrated. Moreover, remarkable enhancement of inhibitory immune receptors mediated crosstalk between skin immune cells was observed in L-LEP lesions. For PBMCs, a high expression level of *APOE* in the HLA-DR^high^FBP1^high^ monocyte subset and the expansion of regulatory T cells were found to be associated with L-LEP. These findings revealed the primary suppressive landscape in the L-LEP patients, providing potential targets for the intervention of intracellular bacteria caused persistent infections.

## Introduction

Infectious diseases caused by bacteria, viruses, fungi, and parasites currently represent the direct cause of 15% of all deaths, globally^1^. Intracellular bacteria are estimated to put approximately one billion individuals at risk, posing a substantial threat to public health^2,3^. Intracellular bacteria have evolved a diverse number of immune escape strategies to reside and proliferate in host cells and establish a persistent or even lifelong infection, among which two common strategies are shared by several pathogens: (1) evasion of host immune recognition; and (2) modulation of the host immune response^4^.

*Mycobacterium leprae* (Mlep), the etiologic agent of leprosy and first identified human pathogenic bacterium, primarily infects the skin and peripheral nerve and can cause irreversible disability and deformities^5–7^. Leprosy was historically prevalent throughout the world and the annual number of newly detected cases globally remains over 200,000, 60% of which are lepromatous leprosy (L-LEP)^8,9^. L-LEP is a disseminated and severe form of the disease and is characterized by diffuse skin lesions and massive proliferation of Mlep in macrophages. In addition, L-LEP represents the main source of infection due to the fact that humans are largely the only reservoir of Mlep^10–12^. Due to the remarkable genomic conservation, despite the global geographic distribution and a history of thousands of years, leprosy has long been recognized as an attractive disease model for investigating the modulation of the human immune response against intracellular bacteria, which leads to persistent infection^13–15^. However, although the increased frequencies of immune cells with an immunosuppressive function, including regulatory T (Treg)^16^, T helper (Th) 22^17^, and FoxP3^+^ γδT cells^18^, have frequently been reported in L-LEP, a comprehensive understanding of the modulation Mlep on host immune response remains unknown. Moreover, the molecular mechanism underlying immuno-compromisation in L-LEP that promotes the proliferation of Mlep has not yet been adequately elucidated.

Single-cell RNA-sequencing (scRNA-seq) provides a powerful tool for resolving cell-type-specific immune responses and has delineated the molecular mechanisms of dysfunction or exhaustion of myeloid and T cells in infectious diseases, including COVID-19 and tuberculosis^19–21^. To characterize the suppressive immune signature of L-LEP at a single-cell resolution, we performed scRNA-seq of both L-LEP lesions and peripheral blood mononuclear cells (PBMCs). The immune landscape of L-LEP was primarily disclosed. In particular, we demonstrated remarkable upregulation of *APOE* in one macrophage subset and exhaustion of CD8^+^ T cells in L-LEP lesions. We also revealed the expansion of Treg cells in L-LEP patients’ PBMCs. These findings shed light on the molecular mechanisms by which intracellular pathogens modulate the host immune response to facilitate their survival and proliferation in host cells.

## Results

### Cohorts and study design

**Fig. 1:**
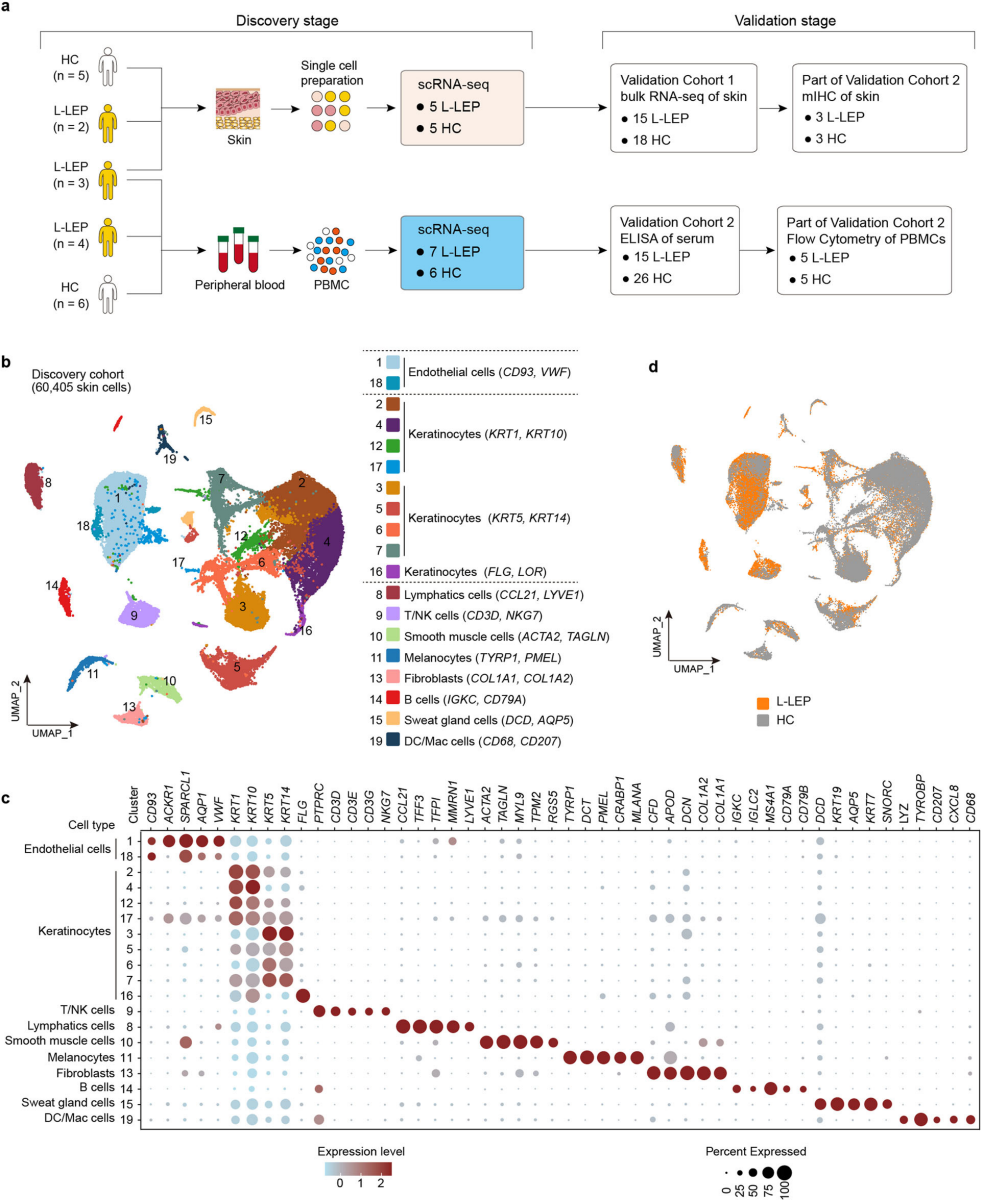
Cohorts definition, study design, and major skin cell types revealed by clustering of scRNA-seq data. **a** Schematic diagram for the cohorts definition and study design. The number of cases and controls for the discovery cohort and each validation cohort were indicated. **b** UMAP visualization of skin scRNA-seq profile of the discovery cohort, discriminative marker genes for each cell type was indicated in parentheses. **c** Dot plot of the top marker genes for the identification of major skin cell types. **d** UMAP plot for skin cells split by patients and HC.

To resolve the cell type-specific immune response in L-LEP, in the discovery cohort, skin lesions from five L-LEP patients and normal skin biopsies from five healthy controls (HC), PBMCs from seven L-LEP patients and six HC, were subjected to scRNA-seq (Fig. 1a). To confirm our findings in the discovery cohort, two validation cohorts were included. Fifteen L-LEP patients and 18 HC were recruited for the bulk RNA-sequencing (bRNA-seq) of lesions and normal skin respectively (validation cohort 1) (Fig. 1a). Validation cohort 2, including 15 L-LEP patients and 26 HC, was used for protein level experiments, i.e., multiple immunohistochemistry (mIHC), enzyme-linked immunosorbent assay (ELISA), and flow cytometry (Fig. 1a).

### Skin immune cells composition revealed by stepwise clustering of scRNA-seq profile

In the discovery cohort, in total 60,405 skin cells from 5 lesions and 5 normal skin biopsies were analyzed and 10 major cell types (keratinocytes, endothelial cells, lymphatic cells, melanocytes, fibroblasts, sweat gland cells, smooth muscle cells, T/Nature killer (NK) cells, B cells, and dendritic cells/macrophages (DC/Mac)) consisting of 19 clusters were identified using a graph-based method. The uniform manifold approximation and projection (UMAP) dimensionality reduction method was used for visualization (Fig. 1b, c; Supplementary Fig. S1a and Table S1). All clusters contained cells from each donor, except that 91.59% of the B cells (Clusters 14) were from patient L-LEP4 and others donors contained very few or no B cells (Supplementary Fig. S1a, b and Table S2). Clusters of L-LEP patients and HC merged well, and no cluster shifting was observed in L-LEP patients compared with HC (Fig. 1d; Supplementary Fig. S1b). Moreover, percentages of cell types in each donor showed no significant differences between L-LEP patients and HC (Supplementary Fig. S1c).

**Fig. 2:**
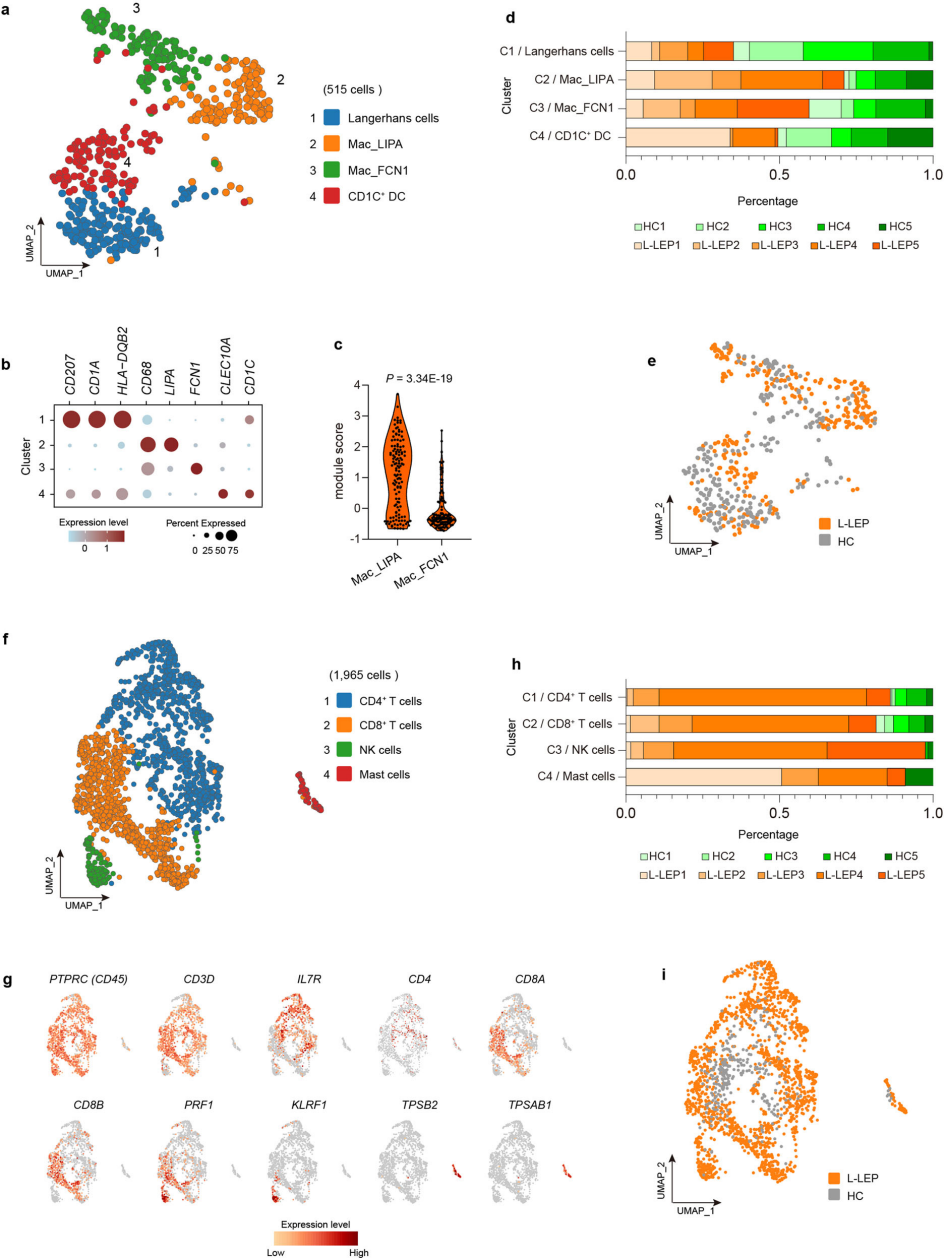
Sub-clustering of the skin DC/Mac and T/NK clusters. **a** UMAP visualization of the sub-clustering of skin DC/Mac cells of the discovery cohort. **b** Dot plot of the top marker genes for the identification of subsets in the skin DC/Mac cluster. **c** Module scores of each macrophage subset calculated using the top ten signature genes of each cluster (genes in Supplementary Fig. S2d). *P* value was calculated using a two-sided unpaired Student’s *t-*test. **d** Donor composition for each cluster obtained in the sub-clustering of skin DC/Mac cells. **e** UMAP plot for skin DC/Mac cells split by patients and HC. **f** UMAP visualization of the sub-clustering of skin T/NK cells of the discovery cohort. **g** UMAP plot of the top marker genes for the identification of subsets in the skin T/NK cluster. **h** Donor composition for each cluster obtained in the sub-clustering of skin T/NK cells. **i** UMAP plot for skin T/NK cells split by patients and HC.

To reveal the composition of the DC/Mac cluster (Cluster 19 in Fig. 1b), a sub-clustering analysis was performed. The sub-clustering of DC/Mac cells identified four subsets, including Langerhans cells (*CD207*, *CD1A*, *HLA-DQB2*), macrophages subset 1 (*CD68*, *LIPA*) (Mac_LIPA), macrophages subset 2 (*CD68*, *FCN1*) (Mac_FCN1) and CD1C^+^ dendritic cells (DC) (*CLEC10A*, *CD1C*)^22^ (Fig. 2a, b; Supplementary Table S1 and Fig. S2a). We also calculated the module score of each macrophage cluster using the top ten signature genes of each cluster, and the results showed that the module scores of two macrophage subsets were significantly different, which validated the annotation of these two clusters (Fig. 2c). Langerhans cells and two macrophages subsets contained cells from each donor, while patient L-LEP2 did not contain any CD1C^+^ DC cells (Fig. 2d, e; Supplementary Table S2 and Fig. S2b). No significant difference in the proportion of each cluster was observed between L-LEP and HC (Supplementary Fig. S2c).

To further analyze the composition of T/NK cells cluster, sub-clustering analysis was also performed, and four subsets were revealed: CD4^+^ T cells (cluster 1) (*IL7R* and *CD4*)^23,24^, CD8^+^ T cells (cluster 2) (*CD8A*, *CD8B*), NK cells (cluster 3) (*KLRF1, PRF1*), and Mast cells (cluster 4) (*TPSB2*, *TPSAB1*) (Fig. 2f, g; Supplementary Table S1 and Fig. S3a). CD4^+^ and CD8^+^ T cells included cells from all donors, while 97.54% of NK cells and 91.04% of mast cells were derived from patients (Fig. 2h, i; Supplementary Table S2 and Fig. S3b). Cell type proportion analysis showed no significant difference between L-LEP and HC (Supplementary Fig. S3c).

In summary, nine skin immune cell subtypes were revealed, including B cells, CD4^+^ T, CD8^+^ T, NK, mast cells, Langerhans cells, Mac_LIPA, Mac_FCN1, and CD1C^+^ DC. Given that B, NK, and mast cells were derived predominantly (> 90%) from patients, further comparative analysis of these cells between L-LEP patients and HC was not conducted.

### *APOE* with the immune regulatory function was remarkably upregulated in Mac_LIPA of L-LEP lesions

**Fig. 3:**
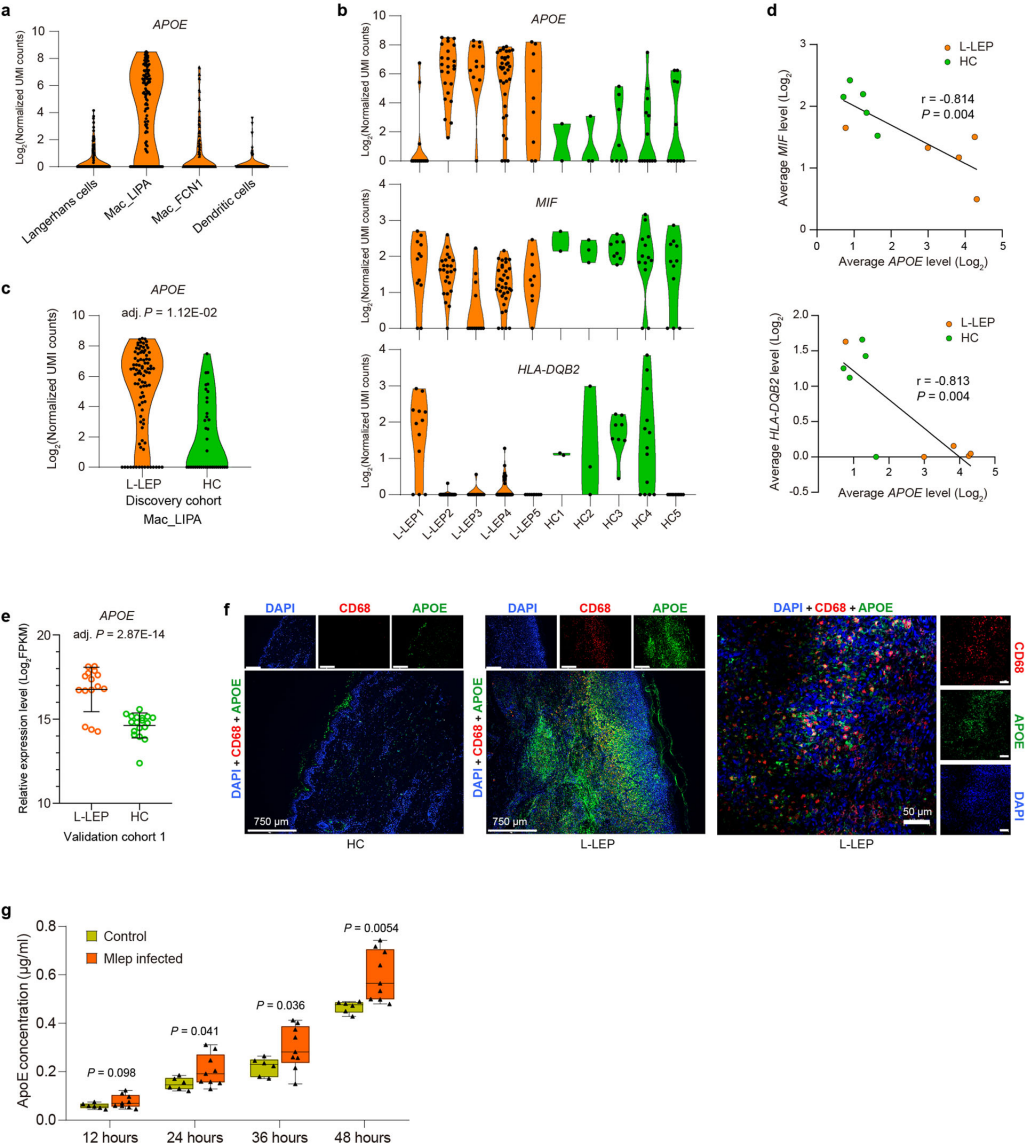
Upregulation of *APOE* with immune modulation function in Mac_LIPA of L-LEP. **a** Expression of *APOE* in each cluster was obtained by the sub-clustering of skin DC/Mac cells. UMI, unique molecular identifier. **b** Expression of *APOE*, *MIF*, and *HLA-DQB2* in Mac_LIPA cells of each sample. **c** Expression of *APOE* in Mac_LIPA cells by cases and controls of the discovery cohort. **d** The average expression level of *APOE* was negatively correlated with *MIF* and *HLA-DQB2* expression, respectively (Pearson’s correlation analysis). Each dot represents a donor. The Pearson correlation coefficient (*r*) and *P* value were indicated. **e** Expression of *APOE* in the skin by cases and controls of validation cohort 1 as determined by bRNA-seq. FPKM, fragments per kilobase of exon model per million mapped fragments. **f** mIHC showing the co-localization of *APOE* and macrophages marker CD68 in L-LEP lesions. The cell nucleus was stained by diamidino-2-phenylindole (DAPI). **g** ApoE protein concentration in the supernatant of human monocyte-derived macrophages infected with/without Mlep. ApoE protein concentration was measured by ELISA at 12, 24, 36, and 48 h after infection with Mlep. *P* value was calculated using a two-sided unpaired Student’s *t* test.

Macrophages represent the major host cells of Mlep in vivo. Compared with three other subsets of DC/Mac, we found that subset Mac_LIPA expressed a significantly high level of *APOE* (Fig. 3a; Supplementary Fig. S2d and Table S1). Analysis of differentially expressed genes (DEGs) between L-LEP patients and HC showed that *APOE* was remarkably upregulated, while *LIPA* expression showed no significant difference in Mac_LIPA of the L-LEP lesions (Fig. 3b, c; Supplementary Table S3). Similar to previous reports that *APOE* can modulate inflammatory and immune responses^25^, we found that in the Mac_LIPA cells of our samples, *APOE* expression was negatively correlated with the expression of the major histocompatibility complex (MHC) II genes *HLA-DQB2* and *MIF*, which encodes a pro-inflammatory cytokine with mycobacteria controlling function (Fig. 3b, d). In validation cohort 1, bRNA-seq also demonstrated a remarkable upregulation of *APOE* in L-LEP (Fig. 3e). High APOE expression and its co-localization with the macrophage marker, CD68, in the L-LEP lesions (validation cohort 2) were further confirmed by mIHC experiment (Fig. 3f).

To investigate whether Mlep infection can induce Apolipoprotein E (ApoE) expression, we infected human monocyte-derived macrophages with Mlep. Our results showed that the concentration of ApoE protein in the supernatant of Mlep-infected macrophages was significantly higher than that of uninfected macrophages, especially at 48 h after infection, indicating that Mlep infection can induce macrophages to upregulate the expression of ApoE (Fig. 3g). Taken together, our findings demonstrated a correlation between the high expression of *APOE* caused by the infection of Mlep and the inhibition of pro-inflammatory and antimicrobial innate immune responses in macrophages.

### CD8^+^ T cells in L-LEP lesions showed an exhausted state

**Fig. 4:**
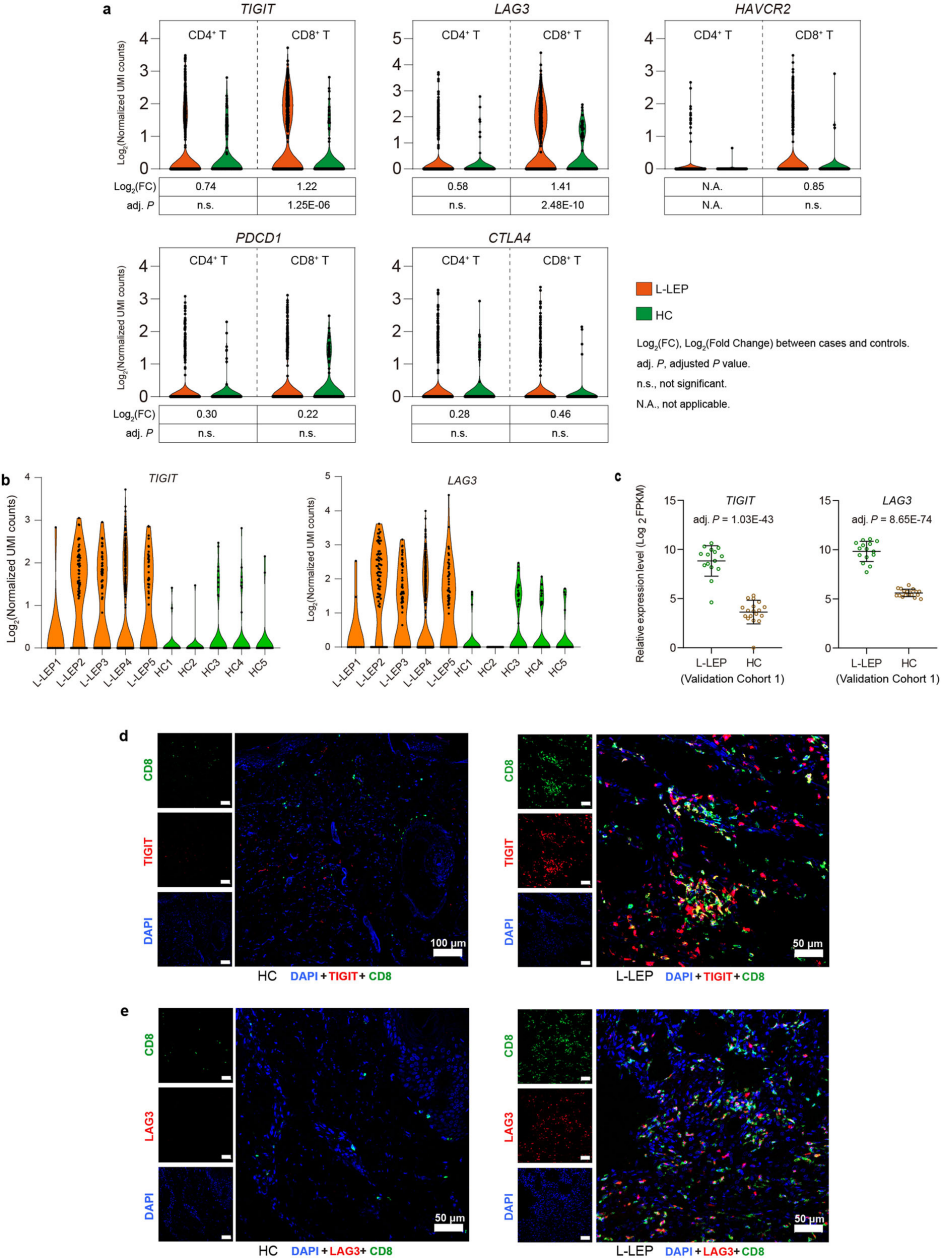
CD8^+^ T cells in L-LEP lesions showed an exhausted state in L-LEP patients. **a** Expressions of *TIGIT*, *LAG3*, *HAVCR2*, *PDCD1*, and *CTLA4* in skin CD4^+^ and CD8^+^ T cells of the discovery cohort. **b** Expressions of *TIGIT* and *LAG3* in the skin CD8^+^ T cells of each sample. **c** Expressions of *TIGIT* and *LAG3* in the skin by cases and controls of validation cohort 1 as determined by bRNA-seq. **d** Co-localization of TIGIT and CD8 in L-LEP lesions. The nucleus was stained by DAPI. **e** Co-localization of LAG3 and CD8 in L-LEP lesions. The nucleus was stained by DAPI.

In addition to T cell receptor signaling, the activation or inhibition of lymphocytes also requires a second signal, the positive co-stimulatory signals or the inhibitory receptor-mediated co-inhibitory signals. During persistent infections, immunosuppression caused by inhibitory receptors has been shown to facilitate the persistence of pathogens in the host^26^. We analyzed the expression of inhibitory receptors (exhaustion markers) *PDCD1* (*PD-1)*, *CTLA-4*, *LAG3*, *TIGIT*, and *HAVCR2* (*TIM-3)* in T cells. Compared with HC, all five exhaustion marker genes showed a trend of increased expression in CD4^+^ and CD8^+^ T cells in L-LEP lesions (Fig. 4a). Notably, in patients’ CD8^+^ T cells, we observed significant upregulation of *TIGIT* and *LAG3* (Fig. 4a, b), implying the exhaustion of CD8^+^ T cells in L-LEP lesions^27,28^. While, in CD4^+^ T cells, no significant difference in the expression level of these five exhaustion markers was observed between L-LEP and HC (Fig. 4a). Moreover, bRNA-seq of validation cohort 1 also demonstrated that *TIGIT* and *LAG3* were remarkably and significantly upregulated in L-LEP (Fig. 4c). Using validation cohort 2, we confirmed the higher expression of TIGIT and the co-localization of TIGIT with CD8^+^ T cells in L-LEP lesions, compared with HC by mIHC (Fig. 4d). Our mIHC results also confirmed the aforementioned findings for LAG3 (Fig. 4e).

To further characterize the state of CD8^+^ T cells in L-LEP patients, we compared the DEGs of CD4^+^ and CD8^+^ T cells. As a result, 251 DEGs that were shared by CD4^+^ and CD8^+^ T cells showed consistent tendencies of expression changes in these 2 types of T cells, while 211 DEGs were CD8^+^ T cell-specific (Supplementary Fig. S3d and Table S3). We performed Gene Ontology (GO) functional enrichment analysis on these 211 CD8^+^ T cells-specific DEGs (Supplementary Table S4). For CD8^+^ T cells-specific DEGs that were downregulated in L-LEP patients, the top significantly enriched GO terms included cell/leukocyte activation, negative regulation of the apoptotic process, and negative regulation of cell death (Supplementary Fig. S3e). These results indicated that CD8^+^ T cells in L-LEP lesions may be dysfunctional in Cell/Leukocyte activation and anti-apoptosis signaling.

Collectively, our results indicated that CD8^+^ T cells in L-LEP lesions were probably in an exhausted state.

### Differential regulation of MHC I and II genes in Langerhans cells of L-LEP patients

**Fig. 5:**
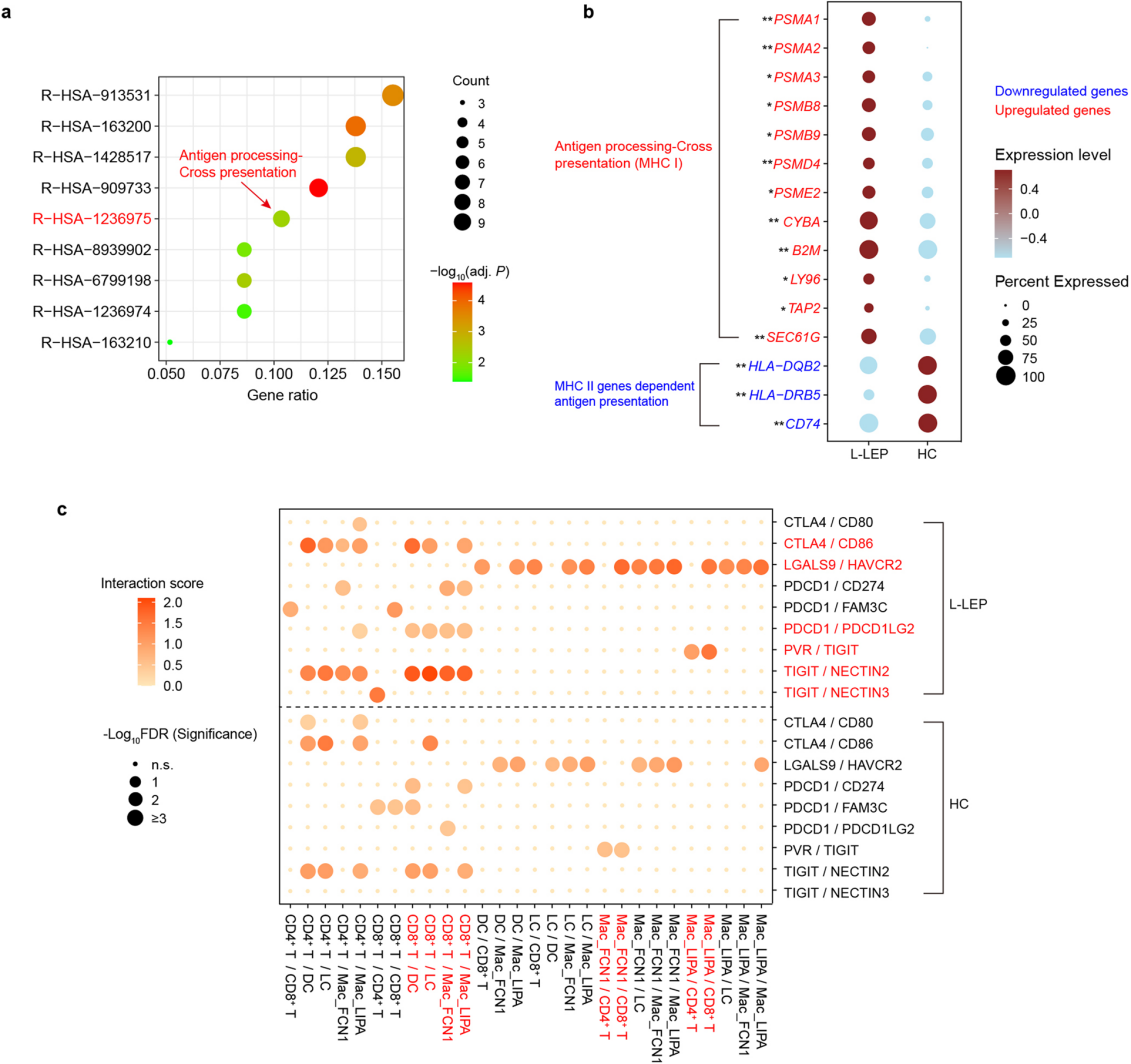
Differential regulation of MHC I and II genes in Langerhans cells of L-LEP patients and the inhibitory receptors mediated interactions between skin immune cells. **a** Functional enrichment analysis (Reactome database) using upregulated DEGs in Langerhans cells of the discovery cohort. The gene ratio represented the ratio of gene number enriched in a pathway to the input DEGs number. **b** Dot plot showing the differential expression of MHC I and II related genes in Langerhans cells of discovery cohort. * and ** indicated a significant difference between L-LEP and HC at *P* < 0.05 and adjusted *P* < 0.05 level, respectively. **c** Bubble chart showing the inhibitory receptors mediated interactions between skin immune cells (discovery cohort). n.s. not significant, FDR false-discovery rate.

Two types of DC, Langerhans cells and CD1C^+^ DC, were identified in our skin scRNA-seq data of the discovery cohort. Analysis on DEGs of CD1C^+^ DC barely found any genes that were relevant to antigen presentation or responses to infection (Supplementary Table S3). Langerhans cells are professional antigen-presenting cells in the skin. In the discovery cohort, skin immune cells that expressed high levels of *CD207*, *CD1A*, and *HLA-DQB2* were identified as Langerhans cells (Fig. 2a, b)^29^. The functional enrichment analysis using upregulated DEGs in L-LEP showed that antigen processing-cross presentation (Reactome database) was significantly enriched (Fig. 5a; Supplementary Table S5). Further analysis found that the upregulation of gene *B2M*, *CYBA*, and proteasome genes (*PSMA1*, *PSMA2*, *PSMD4*, *SEC61G*), which play essential roles in class I MHC antigen presentation^30^, was responsible for the significant enrichment of antigen processing-cross presentation pathway (Fig. 5b; Supplementary Tables S3 and S5). Meanwhile, we observed downregulation of MHC II genes, such as *CD74*, *HLA-DQB2*, and *HLA-DRB5* in L-LEP (Fig. 5b; Supplementary Table S3). These results suggested that MHC I molecule-dependent antigen presentation to CD8^+^ T cells was enhanced, while MHC II molecule-dependent antigen presentation to CD4^+^ T cells was decreased in Langerhans cells of L-LEP lesions.

### Inhibitory receptor-mediated interactions between skin immune cells were enhanced in L-LEP lesions

To detect alterations of intercellular communications in skin immune cells of L-LEP, ligand and receptor interactions analyses on skin immune cells (CD8^+^ T cells, CD4^+^ T cells, CD1C^+^ DC, Mac_LIPA, Mac_FCN1, and Langerhans cells) were performed separately for L-LEP patients and HC of the discovery cohort (Supplementary Table S6). Overall, we observed remarkably enhanced crosstalk in L-LEP lesions mediated by CTLA4/CD86, PDCD1 (PD-1)/PDCD1LG2, LGALS9/HAVCR2 (TIM-3), PVR/TIGIT, TIGIT/NECTIN2, and TIGIT/NECTIN3. (Fig. 5c; Supplementary Table S6). The enhancements of interactions between CD8^+^ T cells and antigen-presenting cells, such as CTLA4/CD86, PDCD1/PDCD1LG2, and TIGIT/NECTIN2, were the most noticeable, which was consistent with the exhausted state of CD8^+^ T cells (Fig. 5c). Interestingly, the PVR/TIGIT interaction was mediated by Mac_FCN1 and T cells in HC, while in L-LEP this interaction was enhanced and presented only between Mac_LIPA and T cells (Fig. 5c), implying the critical role of Mac_LIPA in L-LEP. Collectively, these results demonstrated an association between inhibitory receptor-mediated interactions and immunosuppression in L-LEP lesions.

### The immunosuppression profile of L-LEP patients’ PBMCs

**Fig. 6:**
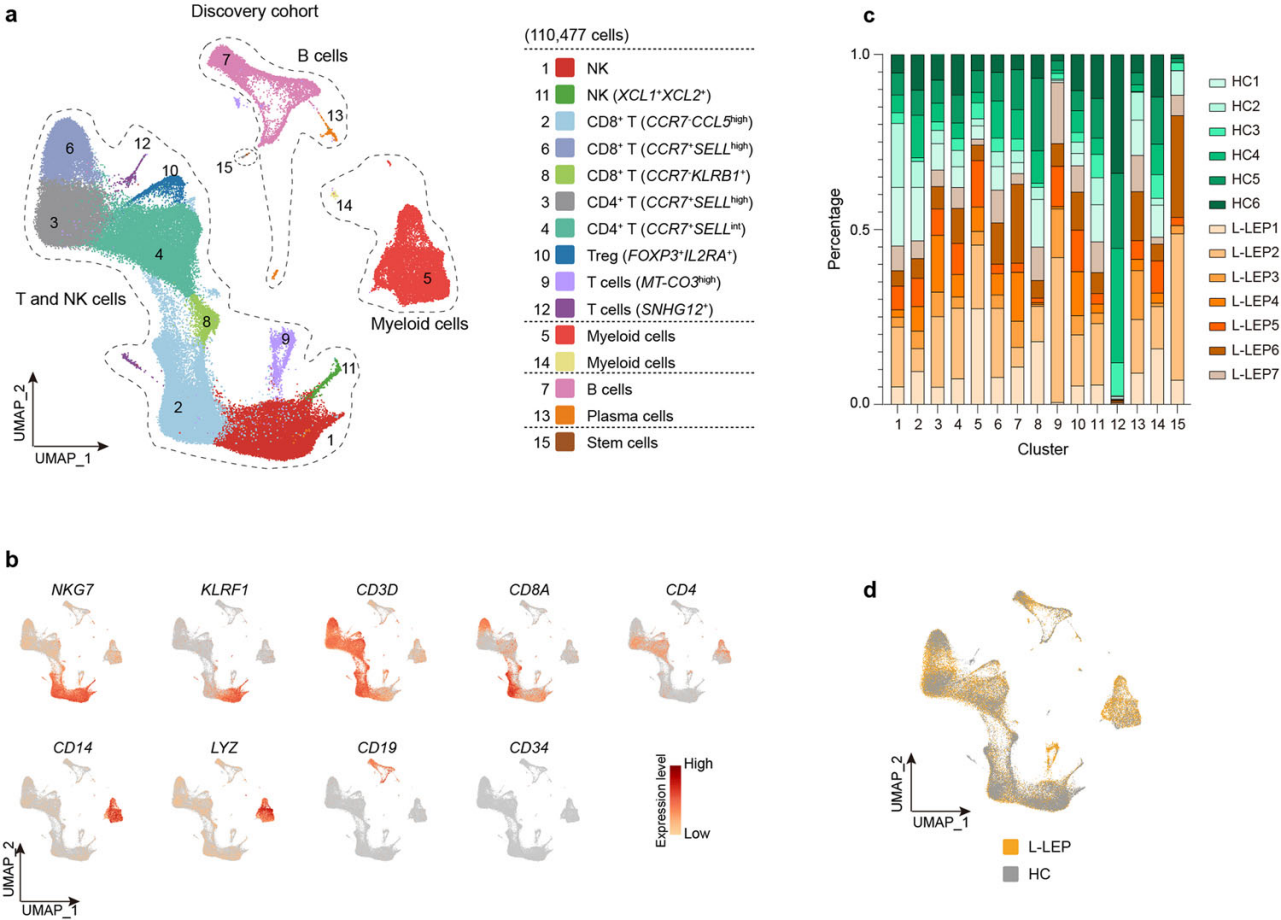
PBMCs composition as revealed by clustering of scRNA-seq data. **a** UMAP visualization of the PBMCs scRNA-seq profile of the discovery cohort. **b** Expressions of major discriminative marker genes for cell types identification of PBMCs. **c** Donor composition for each cluster of PBMCs. **d** UMAP plot for PBMCs split by patients and HC.

In order to investigate whether L-LEP patients’ PBMCs also showed immunosuppression signals, we performed scRNA-seq for the PBMCs of seven L-LEP patients and six HC (discovery cohort). In total, 110,477 cells were analyzed and 15 subsets were identified, including two NK cell subsets, three CD8^+^ T cell subsets, two CD4^+^ T cell subset, Treg subset, two T cell subsets, two myeloid cell subsets, B cells, plasma cells, and stem cells (Fig. 6a, b; Supplementary Fig. S4a, b and Table S1). Composition analysis showed that each cluster contained cells from each donor (Fig. 6c). Clusters of L-LEP patients and HC merged well, and L-LEP patients showed no cluster shifting compared with HC (Fig. 6d; Supplementary Fig. S4c). Comparison of percentage of each cluster between L-LEP and HC showed an only higher percentage of cluster 9 and a lower percentage of cluster 12 in L-LEP (Supplementary Fig. S4d). Cluster 9 was an undefined T cluster that expressed a high level of non-coding gene *AC114760.2* and mitochondrial genes, such as *MT-CO3* (Supplementary Table S1). Cluster 12 was the minimal and undefined T cells cluster which showed specific expression of noncoding gene *SNHG12* (Supplementary Table S1), showing high heterogeneity among HC (Fig. 6c; Supplementary Table S2). Therefore, clusters 9 and 12 were not subjected to further analysis.

**Fig. 7:**
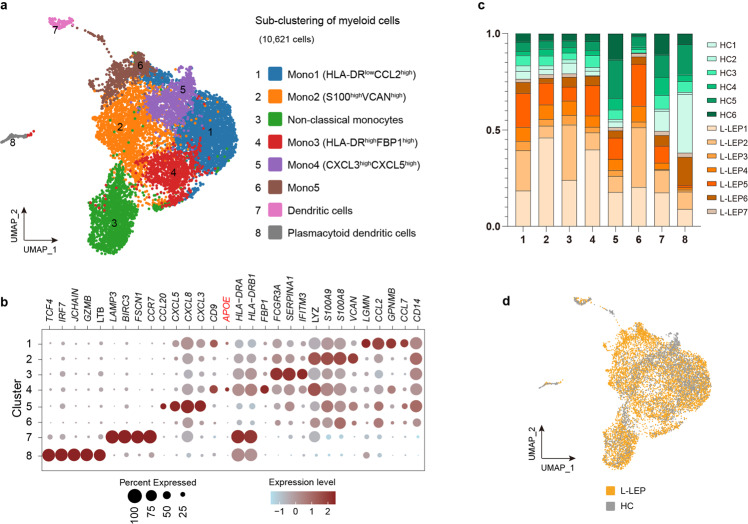
Sub-clustering of myeloid cells of PBMCs. **a** Sub-clustering of myeloid cells in the discovery cohort as shown by a UMAP plot. **b** Dot plot of the top marker genes and the *APOE* gene for myeloid cells subsets of the discovery cohort. **c** Donor composition for each cluster obtained in the sub-clustering of myeloid cells. **d** UMAP plot for myeloid cells split by patients and HC.

Given that myeloid cells are highly heterogeneous^31^, we performed a sub-clustering of myeloid cells. In total, eight clusters were identified, including six monocyte subsets (Mono1-Mono5 and non-classical monocytes), DC and plasmacytoid DC. All clusters were composed of cells from all 13 donors, except that HC2 did not contain any cells of cluster 6 (Fig. 7a–c; Supplementary Fig. S5a). These eight clusters of L-LEP patients and HC merged well, and no cluster shifting was observed between L-LEP patients and HC (Fig. 7d; Supplementary Fig. S5b). Comparison of the percentage of each cluster in PBMCs demonstrated no significant difference between L-LEP and HC (Supplementary Fig. S5c).

**Fig. 8:**
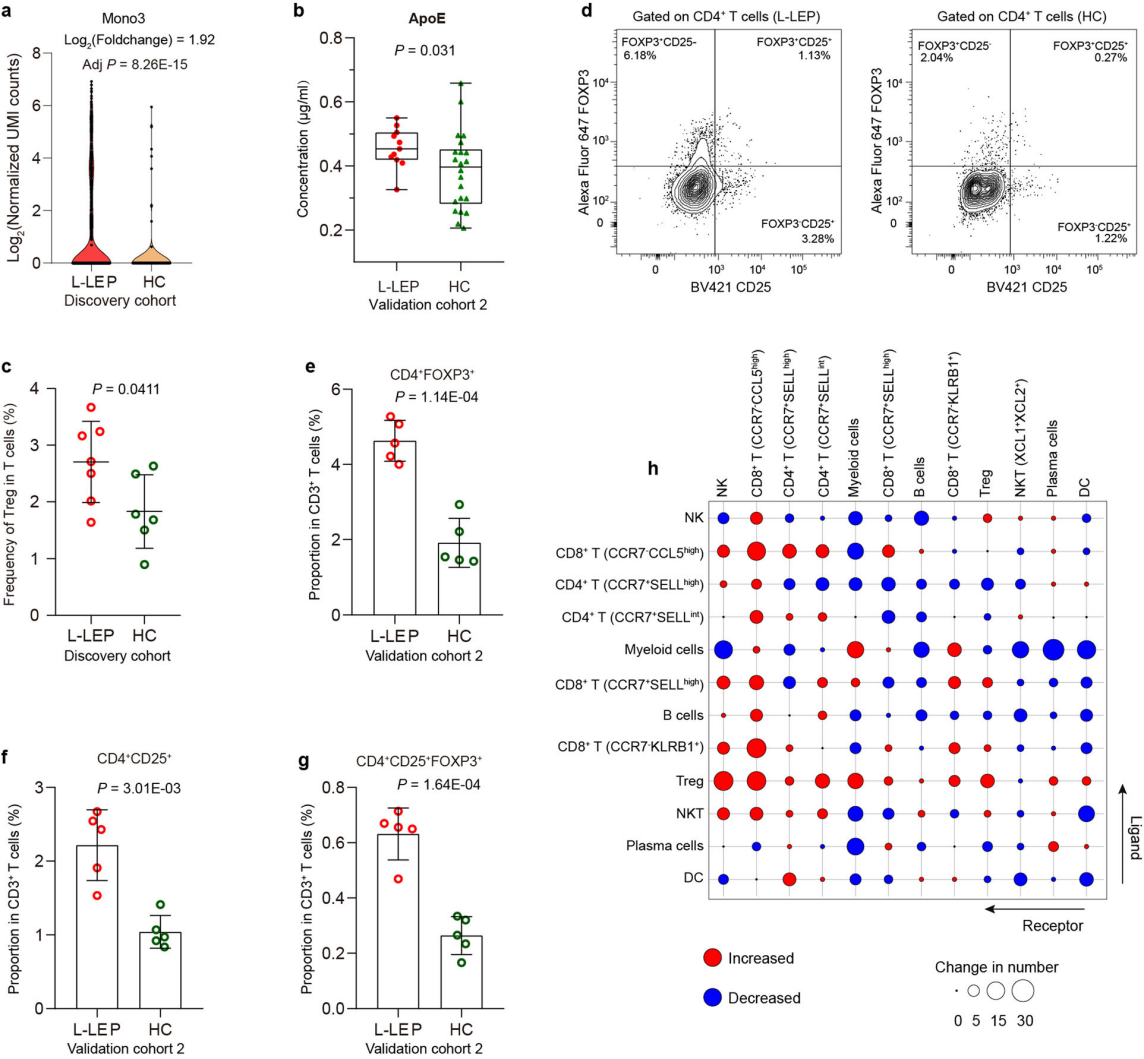
The upregulation of *APOE* in HLA-DR^high^FBP1^high^ monocytes subsets and expansion of Treg in PBMCs of L-LEP patients. **a** Expression of *APOE* in HLA-DR^high^FBP1^high^ monocytes subsets of the discovery cohort. **b** ApoE protein concentration in the serum of L-LEP patients and HC (validation cohort 2) determined by ELISA. *P* value was calculated using a two-sided unpaired Student’s *t* test. **c** Percentage of Treg subset in T cells of the discovery cohort. *P* value was calculated using a two-sided unpaired Student’s *t* test. **d** Representative flow cytometry results for the determination of the percentage of Treg subset in CD3^+^ T cells. **e**–**g** Percentage of CD4^+^ FOXP3^+^, CD4^+^ CD25^+^, and CD4^+^ CD25^+^ FOXP3^+^ Treg subset in CD3^+^ T cells of the validation cohort 2 determined by flow cytometry. *P* value was calculated using a two-sided unpaired Student’s *t* test. **h** The change in numbers of putative ligand-receptor pairs in PBMCs of L-LEP patients compared with HC indicated by dot plots (discovery cohort).

Interestingly, we found that *APOE* was primarily expressed by the Mono3 (HLA-DR^high^FBP1^high^) subset (Fig. 7b; Supplementary Table S1), in which a significant upregulation of *APOE* was observed in L-LEP patients (Fig. 8a). Flow cytometry also confirmed the upregulation of *APOE* in this monocyte subset at the protein level (Supplementary Fig. S6a, b). The serum of L-LEP patients also showed a significantly higher concentration of ApoE protein than HC (validation cohort 2) (Fig. 8b). Considering the aforementioned findings on *APOE*, our results suggested that the Mono3 (HLA-DR^high^FBP1^high^) monocyte subset might be involved in the pathogenesis of Mlep infection.

In T cells, we observed a profound expansion of Treg (*FOXP3*^+^*IL2RA* (*CD25*)^*+*^) in L-LEP patients (Fig. 8c). Further flow cytometry experiments confirmed that the proportions of CD4^+^ FOXP3^+^, CD4^+^ CD25^+^, and CD4^+^ CD25^+^ FOXP3^+^ Treg cells in the patients’ PBMCs were all significantly higher than that of HC (validation cohort 2) (Fig. 8d–g; Supplementary Fig. S6c). We also performed ligand and receptor interaction analysis of PBMCs subsets for L-LEP patients and HC separately. We found that Treg cells showed remarkably increased interactions with other cell types (Fig. 8h; Supplementary Table S7). In summary, high expression of *APOE* in HLA-DR^high^FBP1^high^ monocyte subsets and the expansion of Treg cells might contribute to the immunosuppression of PBMCs in L-LEP patients.

## Discussion

Investigating the modulation of the host immune response induced by intracellular bacteria is critical to understanding the intrinsic mechanisms of a persistent infection, which is of great significance for improving disease diagnosis, intervention, and prophylactics. Although a large number of leprosy susceptibility genes have been discovered^32^, the knowledge of how Mlep survives and proliferates in macrophages by modulating the patients’ immune responses remains limited. In this study, we present a primary immune landscape of the host against Mlep in L-LEP by single-cell transcriptomic analysis of skin lesions and PBMCs, clarifying part of the molecular mechanism by which Mlep escapes the immune response to reside and proliferate within host cells.

Several general mechanisms for the modulation of the host immune response, including autophagy inhibition, cytokine inhibition, and the most common mechanism induction of Tregs, are shared by various intracellular bacteria^4^. However, some important issues remain unresolved, such as the precise cell subtypes affected by pathogens, the host susceptibility genes that interact with pathogens, and the intercellular crosstalk between immune cells. Recently, the scRNA-seq data of four leprosy lesions were published in a study focusing on the development and application of a second-strand synthesis-based scRNA-seq technique in human inflamed skin, and the immunopathogenesis of leprosy was not indicated, except for the identification of macrophages, Langerhans cells, and T cell subsets^29^. The first scRNA-seq study of tuberculosis demonstrated the alteration of the PBMCs composition caused by depletion of a cytotoxic NK cell subset and exhaustion of monocyte subsets in PBMCs, revealing *Mycobacterium tuberculosis* (Mtb) mediated suppression of specific immune subsets^19^. Our current study also revealed an alteration of the PBMCs composition in L-LEP, the expansion of the Treg cell subset. Furthermore, our findings demonstrated the role of *APOE* in the dysfunction of macrophages and the exhaustion of CD8^+^ T cells featured by *TIGIT* and *LAG3* in the lesions of L-LEP, respectively.

ApoE, a multifunctional protein expressed by various cells including macrophages, plays important roles in lipoprotein metabolism and immunomodulation^25,33^. Recently, a scRNA-seq study of leprosy reported the enrichment of the macrophage subset (TREM2 macrophages) exhibiting a high level of *APOE* in L-LEP lesions^34^, but the potential pathogenic function of *APOE* in L-LEP remained to be clarified. Here, we showed that *APOE* was predominantly expressed by the Mac_LIPA subset and was remarkably upregulated in L-LEP lesions. Previous studies reported that exposure of human ApoE receptors-expressing mouse macrophages to ApoE suppressed the pro-inflammatory cytokines TNF-α, etc.^35^. Zhu et al. also demonstrated that exogenous ApoE inhibits the induction of pro-inflammatory cytokines IL-6, IL-1β, and TNF-α through TLR3- and TLR4-mediated macrophage activation^36^. More interestingly, ApoE deficiency was reported to result in increased MHC II molecules expressions on antigen-presenting cells and then caused enhanced CD4^+^ T cells activation^37,38^. In line with these previously reported findings, our study here revealed the negative correlation of *APOE* with *MIF* and MHC II gene *HLA-DQB2* in Mac_LIPA. The Macrophage Migration Inhibitory Factor, encoded by *MIF*, is a pro-inflammatory cytokine and was reported to play a critical role in the control of Mtb by macrophages^39^. Moreover, our in vitro experiments also supported that Mlep infection could induce ApoE expression in macrophages. We also demonstrated that the *APOE* gene was specifically expressed by an HLA-DR^high^FBP1^high^ monocyte subset and *APOE* was significantly upregulated in this monocyte subset of L-LEP, suggesting that this subset might belong to monocytes that are specifically responsive to Mlep. Collectively, our findings suggested that the suppressed immune response in Mac_LIPA may be attributable to the upregulation of *APOE* that was induced by Mlep infection, indicating the immunomodulatory role of *APOE* in leprosy.

The observation of suppressive CD8^+^ T cell in L-LEP lesions with mysterious molecular mechanisms has been previously reported^13^. Here, we reported a profound upregulation of *TIGIT* and *LAG3* in the CD8^+^ T cells of L-LEP lesions. *TIGIT*, as a reliable marker of T cell exhaustion^27,28^, was an inhibitory immune receptor, which could suppress T cell responses directly or indirectly by inducing tolerogenic antigen-presenting cells^40,41^, and has been shown to be highly expressed on viral-infected T cells^42–44^. Similarly, the expression of *LAG3*, another exhaustion marker, was also reported to be elevated in T cells infected by various viruses, such as herpes simplex virus 1, human immunodeficiency virus, and lymphocytic choriomeningitis virus^45–47^. And an increased expression of *LAG3* in lesions of multibacillary leprosy compared to paucibacillary leprosy was also reported recently^48^. Nevertheless, to the best of our knowledge, little is known about the roles of *TIGIT* in chronic bacterial infection. Furthermore, the enhanced interactions of CD8^+^ T cells with antigen-presenting cells, such as CTLA4/CD86, PDCD1/PDCD1LG2, and TIGIT/NECTIN2, suggested the negative regulation of CD8^+^ T cells responses in L-LEP. Our findings suggested that the upregulation of *TIGIT* and *LAG3* in CD8^+^ T cells contributed to the anergy of CD8^+^ T cells in L-LEP lesions, preliminarily revealing the molecular mechanisms of CD8^+^ T cell-mediated immunosuppression in L-LEP.

We observed obvious expansion of Treg cells in PBMCs of L-LEP patients, which was consistent with other studies^16,49^. Moreover, we observed a remarkable increase of communications between Tregs and another cell type, which may be an important way for Treg to exert their immunosuppressive functions in L-LEP patients’ PBMCs. However, no further immunosuppressive signals in L-LEP patients were revealed by our studies of PBMCs, suggesting that the modulation of Mlep on host immune response was mainly restricted to the skin lesions of infection.

With the emergence of antibiotic-resistant and untreatable strains, the development of novel approaches for infectious disease treatment has gained increasing attention^50–53^. One promising approach is immunomodulatory therapy that can enhance host antimicrobial immunity through immunomodulators, including host antimicrobial peptides, as well as agonists of Toll-like receptors and nucleotide-binding and oligomerization domain-like receptors^50,52,53^. However, the application of these general immunity-enhancing modulators can also result in harmful side effects, such as inflammatory tissue damage^50,52^. The discovery of immunosuppressive mechanisms in specific cell subsets in persistent infection provides novel targets for the development of precise immunomodulators with minimum harmful side effects. For example, antagonists targeting *APOE* in macrophages and *TIGIT* and *LAG3* in CD8^+^ T cells may represent promising therapeutic targets for the inhibition of Mlep proliferation in L-LEP.

We acknowledge the limitations associated with the present study. Th subsets have been reported to be involved in the development of leprosy and its clinical subtypes^11^. The study by Hughes et al. identified Th17 subsets in leprosy skin biopsies using the *RORC* gene marker via a second-strand synthesis-based scRNA-Seq technique^29^. In contrast, in our study, we failed to detect *RORC* and the low number of skin immune cells in the discovery cohort also made us unable to further sub-cluster CD4^+^ and CD8^+^ T cells. This led to an insufficient dissection of the transcriptomic signatures of Th cells subsets.

Collectively, we presented the transcriptomic landscape of L-LEP at a single-cell resolution, revealing the immune signatures of disease-associated and immunocompromised monocyte, T cell, and macrophage subsets. These cell subsets and their respective genes with immunosuppressive functions represented potential targets for the prevention and treatment of L-LEP and other intracellular bacteria-caused persistent infectious diseases.

## Materials and methods

### Sample information of the patients and HC

Leprosy was diagnosed according to clinical manifestations, acid-fast staining, and histopathological examination by dermatologists. And the pathogen Mlep was identified by a previously described fluorescent quantitative polymerase chain reaction method^54^. Patients’ information was provided in Supplementary Table S8. Control PBMCs samples were provided by healthy donors and the control skin biopsies were obtained from fractured patients without the infectious or immune-related disease. All subjects provided written informed consent, and this study was approved by the institutional ethics committee of Shandong Provincial Institute of Dermatology and Venereology.

The discovery cohort contained nine L-LEP patients and 11 HC. The validation cohort 1 included 15 L-LEP patients and 18 HC. The validation cohort 2 included 15 L-LEP patients and 26 HC.

### Single-cell preparation, RNA sequencing, and data analysis

Density gradient centrifugation was used to isolate PBMCs using a lymphocyte separation solution (MD pacific Biotechnology Co., Ltd.). Before the barcode labeling of single cells, CD45^+^ PBMCs were sorted using flow cytometry (FACSAria Fusion, BD).

Skin biopsy specimens were disassociated with Dispase II (Sigma) to separate the epidermis and dermis. The minced epidermis was further digested with 0.25% Trypsin-EDTA (Gibco) for 30 min and filtered with a 70 μm cell strainer (Falcon). The dermis was digested with 1 mg/mL Collagenase P (Sigma-Aldrich) and 100 μg/mL DNase I (Sigma-Aldrich) for 50 min and filtered using a 70 μm cell strainer (Falcon).

Barcode labeling of the single cells and library construction were performed using the 10× chromium system (10× genomics). The constructed library was sequenced on an Illumina NovaSeq 6000 System. For each samples dataset, raw sequencing data were aligned and quantified using the CellRanger pipeline (version 3.0.1, 10× Genomics) to the GRCH38 human reference genome. The gene expression matrix was then processed and analyzed by Seurat (version 3.2.0) R (version 3.6.3) package^55^, and cells with total unique molecular identifier numbers fewer than 500 or detected with fewer than 200 genes or higher than 10,000 genes or more than 10% mitochondrial reads or more than 10% red cell reads were removed. Cells predicted to be potential cell doublets using the Python Scrublet package (version v0.2)^56^ were also excluded.

For each sample dataset of filtered gene-barcode matrix, top 2000 highly variable genes were used for the subsequent principal component analysis (PCA) to reduce dimensionality by “RunPCA” function. Then UMAP was performed on the top 30 principal components for visualizing the cells using “RunUMAP” function. Cluster marker genes were identified by applying differential gene expression testing, which was performed using “FindAllMarkers” functions with a filter condition of log_2_ (Foldchange) (log_2_FC) > 0.25 and adjusted *P* values < 0.05. In the sub-clustering of skin DC/Mac cluster, skin T/NK cluster, and PBMCs’ myeloid cells, we found some potential doublets, which have been removed and not included in further analysis. DEGs analysis between the cases and controls were performed using the Likelihood-ratio test method of “FindMarkers” function and then filtered with the condition |log_2_FC| > 0.585 and adjusted *P* values < 0.05. And the “AddModuleScore” function with default parameters was used to calculate module scores. The functional enrichment analysis was performed using g:profiler software^57^.

### Ligand–receptor analysis

We used a ligand–receptor pairs list from CellPhoneDB to identify the intercellular ligand-receptor interactions (https://raw.githubusercontent.com/Teichlab/cellphonedb-data/master/data/interaction_input.csv). To infer potential ligand-receptor interactions between two cell types, R package scTHI (version 1.0.0) was used to perform cell–cell communication analysis. Single-cell transcriptomic data of cells annotated as skin immune cells (including CD8^+^ T cells, CD4^+^ T cells, CD1C^+^ DC, Mac_LIPA, Mac_FCN1, and Langerhans cells) and cells of 14 PBMCs clusters (cluster 15 which was identified as stem cells was not included) were applied for cell–cell interaction analysis. We defined the ligand-receptor score as the mean of the average log-normalized expression of the receptor gene in one cell type and the average log-normalized expression of the ligand gene in a second cell type. The significance of the interaction is then evaluated using a random permutation of the cell types. After 1000 times permutation, the *P* value and false discovery rate (FDR) were calculated by background distribution of ligand-receptor score, the ligand-receptor pairs which FDR < 0.05 were preserved. Ligand-receptor analyses on skin cells and PBMCs, or on L-LEP and HC cells were either performed separately.

### Bulk RNA sequencing and data analysis

The total RNA of the skin biopsy specimens was extracted using a miRNeasy Mini Kit (QIAGEN) in accordance with the manufacturer’s instructions. Library construction was prepared using a TruSeq^®^ RNA LT Sample Prep Kit v2 (Illumina). Constructed libraries were sequenced using a HiSeq3000 (Illumina) sequencer. Quality control of the RNA data was performed using FastQC. Clean reads were mapped to the human reference genome (hg19) using STAR. The negative Binomial Wald test was used to test the differential expression of genes between the cases group and the controls group using DESeq2 package. Differential expression analysis was performed using DESeq2 software. Genes with |log_2_FC| > 1 and FDR value < 0.05 were defined as DEGs.

### mIHC

The skin biopsy was fixed in neutral buffered formalin and embedded in paraffin. Endogenous peroxidase of the paraffin-embedded tissue sections was blocked by 3% H_2_O_2_ and non-specific binding was blocked by 5% bovine serum albumin blocking buffer (Solarbio) after citrate antigen retrieval. The slides were incubated with primary antibodies overnight at 4 °C, followed by incubation with the secondary antibodies, and color development was performed using a Four-color multiple fluorescent immunohistochemical staining kit (Absin) in accordance with the manufacturer’s instructions. The sections were imaged using the EVOSTM FL Auto 2 Imaging System (Thermofisher) or LSM 980 confocal microscope (ZEISS).

### ELISA

Serum samples were centrifuged at 1000× *g* for 5 min to remove the insoluble components. The concentration of the targets proteins was measured using the Human Apolipoprotein E ELISA Kit (APOE) (abcam) in accordance with the manufacturer’s instructions.

### Induction of apolipoprotein E in macrophages by Mlep infection

Human CD14 MicroBeads (Miltenyi Biotec) were used to purify monocytes from PBMCs. CD14^+^ monocytes were differentiated into macrophages by M-CSF (50 ng/mL) (R&D Systems) for seven days in complete medium, RPMI 1640 medium (Gibco) supplemented with 10% fetal bovine serum (Gibco), and 1% Penicillin–Streptomycin (Gibco). After resting in a fresh complete medium for 2 days, macrophages were infected with/without Mlep with MOI = 10. ApoE protein concentration in the cell culture supernatant was measured by ELISA at the time point of 12, 24, 36, and 48 h after infections. And The Human Apolipoprotein E ELISA kit (Abcam) was used.

### Flow cytometry

For analysis of Treg cells, PBMCs were incubated with surface antibodies (CD3, CD4, and CD25) in PBS for 15 min at room temperature. For staining of intracellular FOXP3, cells were fixed and permeabilized with the Transcription Factor Buffer Set (BD Biosciences), followed by antibody incubation for 60 min at 4 °C.

For analysis of APOE in monocyte subsets, before staining with surface antibodies (CD3, CD19, CD14, HLA-DR, and CD9) in PBS for 15 min at room temperature, PBMCs were cultured overnight followed by treating with Brefeldin A (Biolegend) for 4 h. The intracellular staining of the APOE method was the same as that of FOXP3 mentioned above. Data were acquired on the FACSAria Fusion flow cytometer (BD Biosciences) and analyzed with the FlowJo software (BD Biosciences).

### Antibodies

APC Mouse Anti-Human CD45 (Clone HI30), APC-H7 Mouse Anti-Human CD3 (Clone SK7), BV510 Mouse Anti-Human CD19 (Clone SJ25C1), BV786 Mouse Anti-Human CD14 (Clone M5E2), BV421 Mouse Anti-Human HLA-DR (Clone G46-6), and BV605 Mouse Anti-Human CD4 (Clone RPA-T4) used for flow cytometry were purchased from BD Biosciences. Alexa Fluor 647 Mouse Anti-Human FoxP3 (206D), BV421 anti-human CD25 (BC96), PE anti-Apo E Antibody (E6D7) and PerCP/Cyanine5.5 anti-human CD9 (1119a) were purchased from BioLegend. Primary antibodies CD68 (D4B9C) XP^®^ Rabbit mAb (Cell Signaling Technology, CST), Anti-Apolipoprotein E antibody [D6E10] (Abcam), TIGIT (E5Y1W) XP^®^ Rabbit mAb (CST), LAG3 (D2G4OTM) XP^®^ Rabbit mAb (CST), and CD8α (C8/144B) Mouse mAb (CST) were used for mIHC.

## Supplementary information

Supplementary Figures

Supplementary Table S1

Supplementary Table S2

Supplementary Table S3

Supplementary Table S4

Supplementary Table S5

Supplementary Table S6

Supplementary Table S7

Supplementary Table S8

## Data Availability

All data used in this study have been deposited in the GSA (Genome Sequence Archive in BIG Data Center, Beijing Institute of Genomics, Chinese Academy of Sciences), under the accession number PRJCA003909, PRJCA003879, and PRJCA002557. Main R code for the analysis and necessary data are available at GitHub.
