# Supplementary material for: The immune-suppressive landscape in lepromatous leprosy revealed by single-cell RNA sequencing
Source: Cell Discov. 2022 Jan 11;8:2. doi: 10.1038/s41421-021-00353-3 (PMC8748782; doi:10.1038/s41421-021-00353-3)
Supplement: Supplementary file 1 — Supplementary Figures [file 41421_2021_353_MOESM1_ESM.pdf]

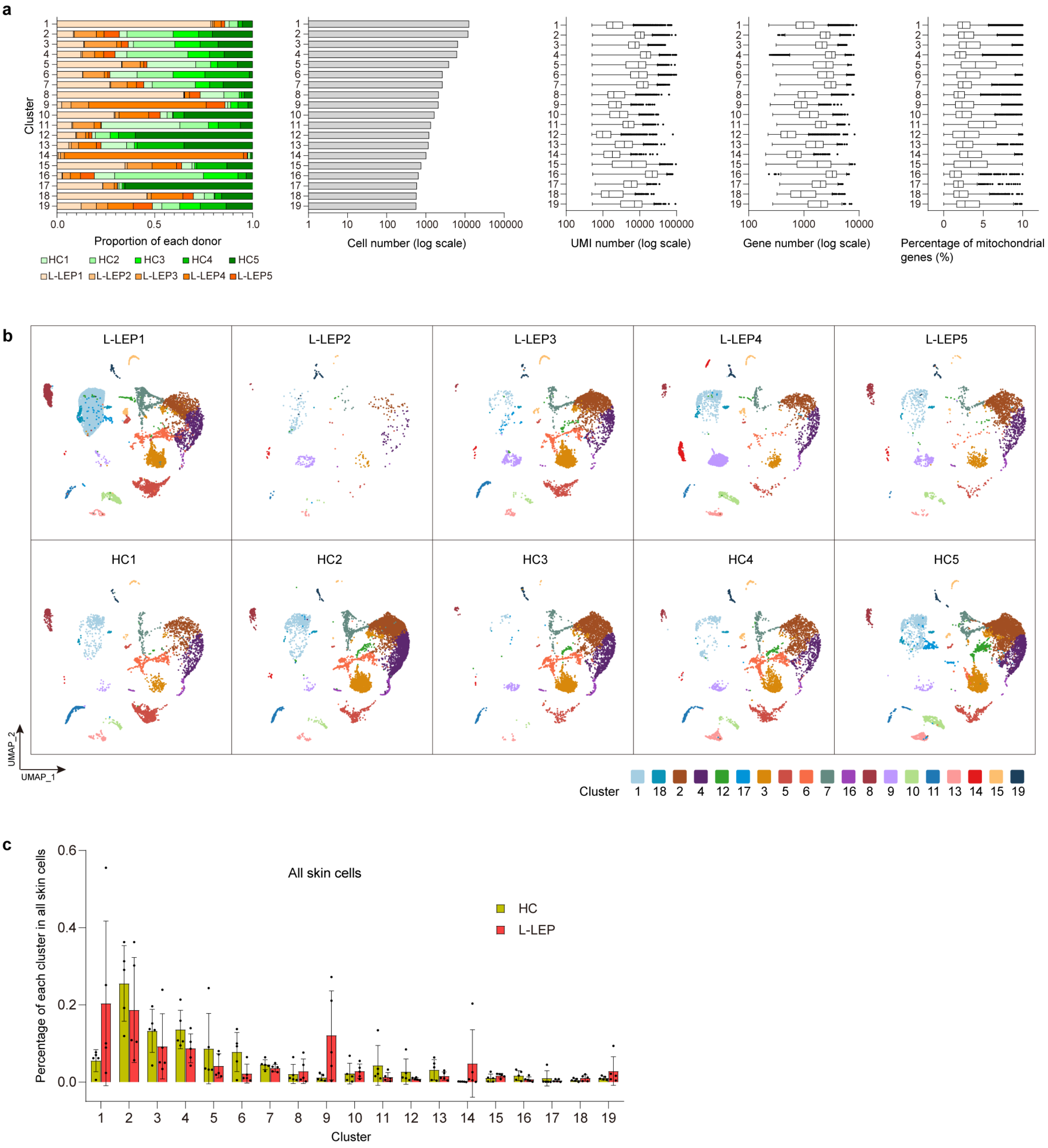
a
b
c
Cluster
Proportion of each donor
Cell number (log scale)
UMI number (log scale)
Gene number (log scale)
Percentage of mitochondrial genes (%)
HC1 HC2 HC3 HC4 HC5
L-LEP1 L-LEP2 L-LEP3 L-LEP4 L-LEP5
L-LEP1
L-LEP2
L-LEP3
L-LEP4
L-LEP5
HC1
HC2
HC3
HC4
HC5
UMAP_1
UMAP_2
Cluster 1 18 2 4 12 17 3 5 6 7 16 8 9 10 11 13 14 15 19
All skin cells
HC
L-LEP
Percentage of each cluster in all skin cells
Cluster

Fig. S1 Clustering of the skin scRNA-seq data of the discovery cohort.

a Quality of each cluster of skin cells. The donor composition, cell number, UMI number, gene number and percentage of mitochondrial genes for each cluster were shown. UMI, unique molecular identifier.

b UMAP plot for skin cells split by each sample.

c Comparison of the percentage of each cluster in all skin cells between L-LEP patients and HC. Each dot represented a donor. *P* value was calculated using two-sample Kolmogorov-Smirnov test.

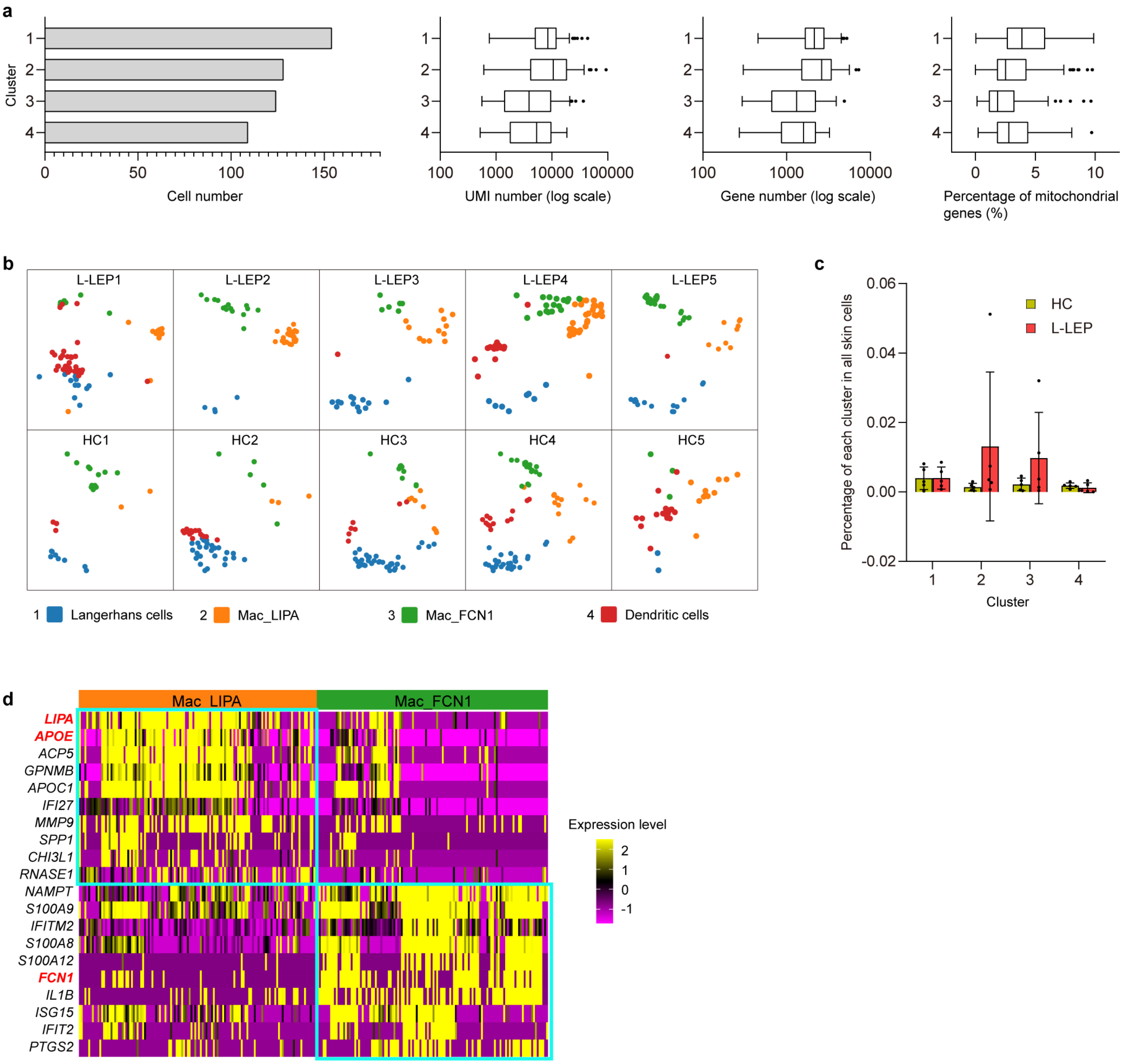

a
Cluster
Cell number
UMI number (log scale)
Gene number (log scale)
Percentage of mitochondrial genes (%)
b
L-LEP1
L-LEP2
L-LEP3
L-LEP4
L-LEP5
HC1
HC2
HC3
HC4
HC5
1 Langerhans cells
2 Mac_LIPA
3 Mac_FCN1
4 Dendritic cells
c
Percentage of each cluster in all skin cells
HC
L-LEP
Cluster
d
Mac_LIPA
Mac_FCN1
LIPA
APOE
ACP5
GPNMB
APOC1
IFI27
MMP9
SPP1
CHI3L1
RNASE1
NAMPT
S100A9
IFITM2
S100A8
S100A12
FCN1
IL1B
ISG15
IFIT2
PTGS2
Expression level

Fig. S2 Sub-clustering of the skin DC/Mac cluster of the discovery cohort.

a Quality of each cluster obtained by the sub-clustering of skin DC/Mac. The cell number, UMI number, gene number and percentage of mitochondria genes for each cluster were shown.

b UMAP plot for skin DC/Mac cells of each sample.

c Comparison of the percentage of each DC/Mac subset in all skin cells between L-LEP patients and HC. Each dot represented a donor. *P* value was calculated using two-sample Kolmogorov-Smirnov test.

d Heatmap showing the expression of signature genes in two macrophage subsets.

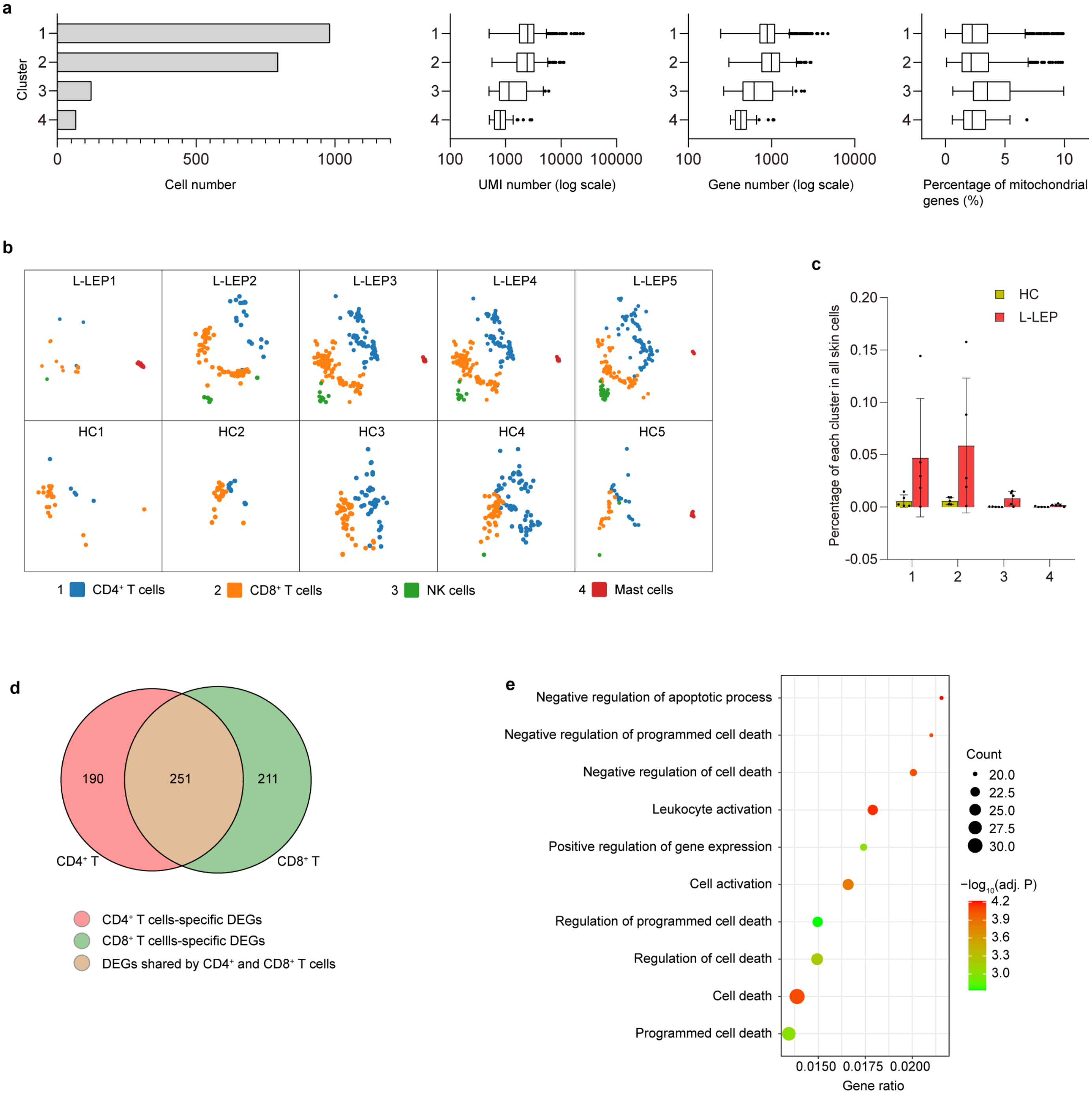
a
Cluster
Cell number
UMI number (log scale)
Gene number (log scale)
Percentage of mitochondrial genes (%)
b
L-LEP1
L-LEP2
L-LEP3
L-LEP4
L-LEP5
HC1
HC2
HC3
HC4
HC5
1 CD4⁺ T cells
2 CD8⁺ T cells
3 NK cells
4 Mast cells
c
Percentage of each cluster in all skin cells
HC
L-LEP
d
190
251
211
CD4⁺ T
CD8⁺ T
CD4⁺ T cells-specific DEGs
CD8⁺ T cellls-specific DEGs
DEGs shared by CD4⁺ and CD8⁺ T cells
e
Negative regulation of apoptotic process
Negative regulation of programmed cell death
Negative regulation of cell death
Leukocyte activation
Positive regulation of gene expression
Cell activation
Regulation of programmed cell death
Regulation of cell death
Cell death
Programmed cell death
Gene ratio
Count
−log10(adj. P)

Fig. S3 Sub-clustering of the skin T/NK cluster of the discovery cohort.

a Quality of each cluster obtained by the sub-clustering of the skin T/NK subset. The cell number, UMI number, gene number and percentage of mitochondrial genes for each cluster were shown.

b UMAP plot for skin T/NK cells of each sample.

c Comparison of the percentage of each T/NK subset in all skin cells between L-LEP patients and HC. Each dot represented a donor. *P* value was calculated using two-sample Kolmogorov-Smirnov test.

d Venn diagram showing the comparison of DEGs between $CD4^+$ and $CD8^+$ T cells.

e Gene Ontology functional enrichment analysis (Biological process) on $CD8^+$ T cells-specific DEGs that were downregulated in L-LEP patients. The gene ratio represented the ratio of gene number enriched in a pathway to the input DEGs number.

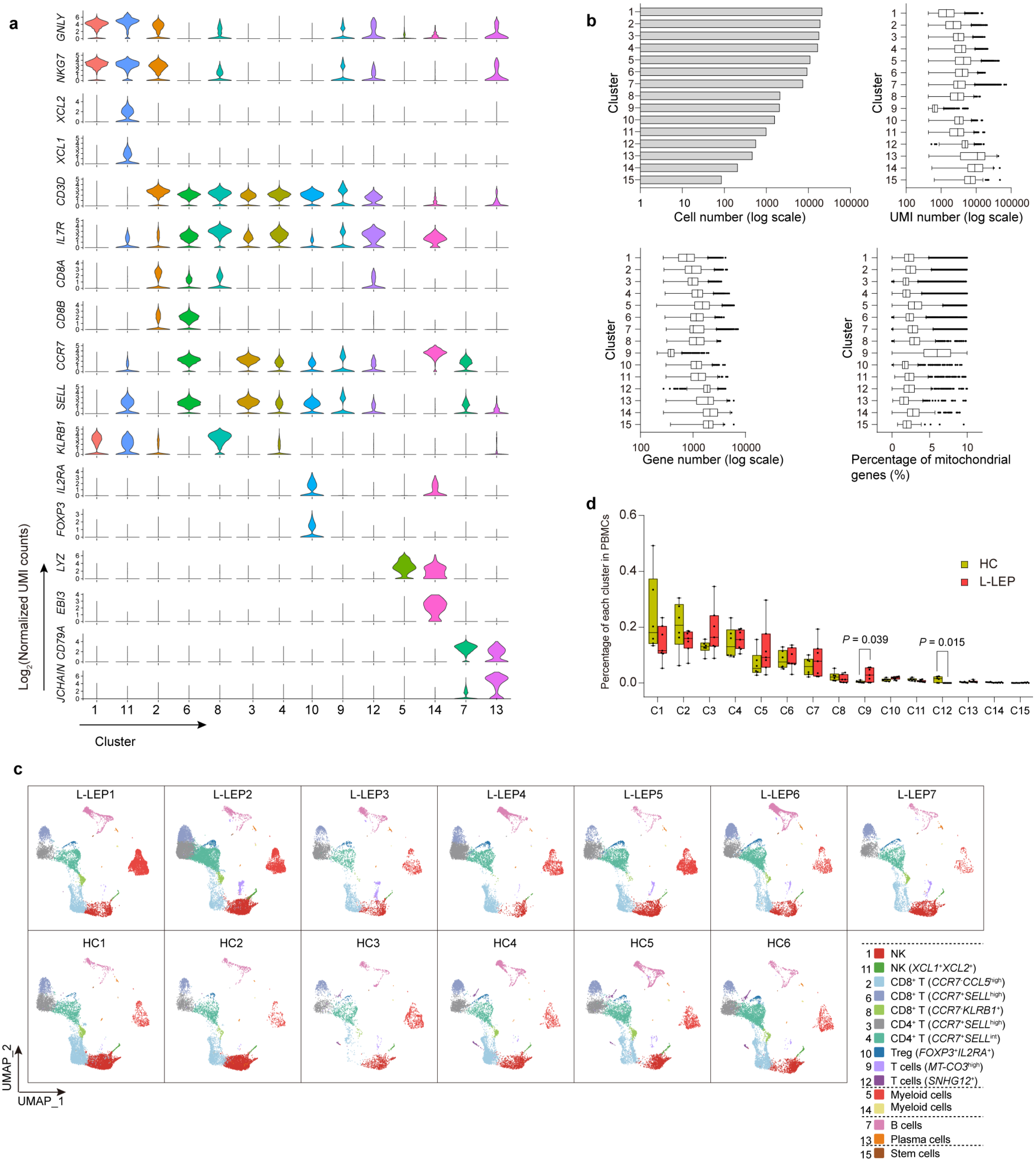
a
GNLY
NKG7
XCL2
XCL1
CD3D
IL7R
CD8A
CD8B
CCR7
SELL
KLRB1
IL2RA
FOXP3
LYZ
EBI3
CD79A
JCHAIN
Log2(Normalized UMI counts)
1 11 2 6 8 3 4 10 9 12 5 14 7 13
Cluster
b
Cluster
Cell number (log scale)
UMI number (log scale)
Gene number (log scale)
Percentage of mitochondrial genes (%)
c
L-LEP1
L-LEP2
L-LEP3
L-LEP4
L-LEP5
L-LEP6
L-LEP7
HC1
HC2
HC3
HC4
HC5
HC6
UMAP_2
UMAP_1
1 NK
11 NK (XCL1+XCL2+)
2 CD8+ T (CCR7-CCL5high)
6 CD8+ T (CCR7+SELLhigh)
8 CD8+ T (CCR7-KLRB1+)
3 CD4+ T (CCR7+SELLhigh)
4 CD4+ T (CCR7+SELLint)
10 Treg (FOXP3+IL2RA+)
9 T cells (MT-CO3high)
12 T cells (SNHG12+)
5 Myeloid cells
14 Myeloid cells
7 B cells
13 Plasma cells
15 Stem cells
d
Percentage of each cluster in PBMCs
HC
L-LEP
P = 0.039
P = 0.015
C1 C2 C3 C4 C5 C6 C7 C8 C9 C10 C11 C12 C13 C14 C15

Fig. S4 Clustering of PBMCs of the discovery cohort.

a Expressions of marker genes for the identification of PBMCs cell types in the discovery cohort indicated by violin plots.

b Quality of each cluster of PBMCs. The cell number, UMI number, gene number and percentage of mitochondrial genes for each cluster were shown.

c UMAP plot for PBMCs of each sample.

d Comparison of the percentage of each cluster in PBMCs between L-LEP patients and HC. Each dot represented a donor. *P* value was calculated using two-sample Kolmogorov-Smirnov test, and *P* value < 0.05 which indicated a significant difference was indicated.

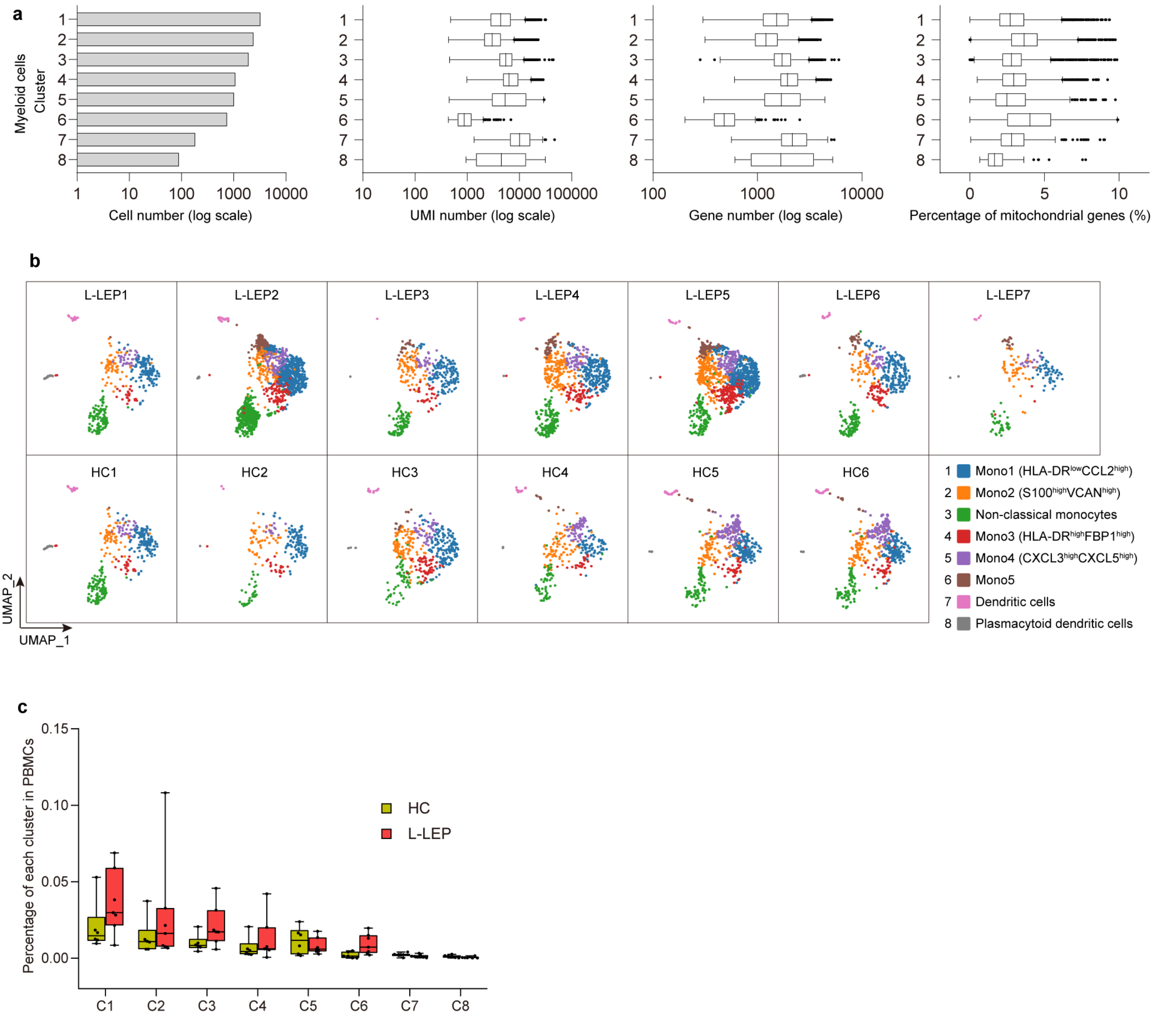
a
Myeloid cells
Cluster
Cell number (log scale)
UMI number (log scale)
Gene number (log scale)
Percentage of mitochondrial genes (%)
b
L-LEP1
L-LEP2
L-LEP3
L-LEP4
L-LEP5
L-LEP6
L-LEP7
HC1
HC2
HC3
HC4
HC5
HC6
UMAP_2
UMAP_1
1 Mono1 (HLA-DR^low CCL2^high)
2 Mono2 (S100^high VCAN^high)
3 Non-classical monocytes
4 Mono3 (HLA-DR^high FBP1^high)
5 Mono4 (CXCL3^high CXCL5^high)
6 Mono5
7 Dendritic cells
8 Plasmacytoid dendritic cells
c
Percentage of each cluster in PBMCs
HC
L-LEP
C1
C2
C3
C4
C5
C6
C7
C8

Fig. S5 Sub-clustering of myeloid cells of PBMCs of the discovery cohort.

a Quality of each cluster obtained by the sub-clustering of myeloid cells. The cell number, UMI number, gene number and percentage of mitochondrial genes for each cluster were shown.

b UMAP plot for myeloid cells of each sample.

c Comparison of the percentage of each myeloid cells subset in PBMCs between L-LEP patients and HC. Each dot represented a donor. *P* value was calculated using two-sample Kolmogorov-Smirnov test.

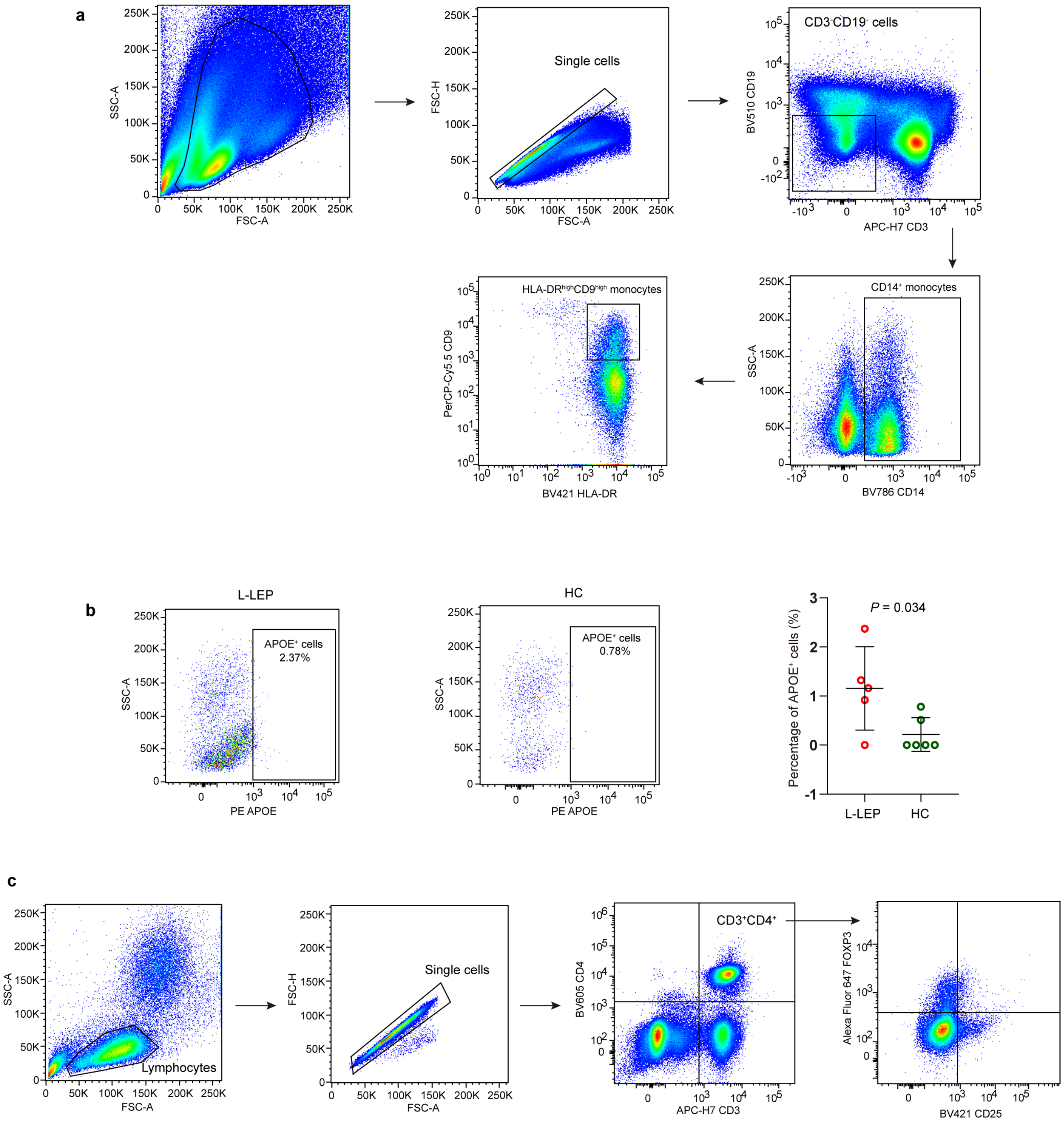
a
Single cells
CD3⁻CD19⁻ cells
CD14⁺ monocytes
HLA-DRhighCD9high monocytes
SSC-A
FSC-A
FSC-H
BV510 CD19
APC-H7 CD3
BV786 CD14
BV421 HLA-DR
PerCP-Cy5.5 CD9
b
L-LEP
HC
APOE⁺ cells 2.37%
APOE⁺ cells 0.78%
PE APOE
P = 0.034
Percentage of APOE⁺ cells (%)
c
Lymphocytes
Single cells
CD3⁺CD4⁺
BV605 CD4
Alexa Fluor 647 FOXP3
BV421 CD25

Fig. S6 Flow cytometry analysis for the HLA-DR^{high}FBP1^{high} monocytes subset and Treg cells in PBMCs.

a Gating strategy for the HLA-DR^{high}CD9^{high} monocytes subset. CD3 and CD19 double negative cells were gated on CD14. CD3^{-}CD19^{-}CD14^{+} monocytes were further gated on CD9 and HLA-DR. Due to the unavailability of an antibody against FBP1 that is suitable for flow cytometry, we used CD9 (Fig. 7b) as a marker for the gating of this monocyte subset.

b Representative and statistical results of APOE expression in the HLA-DR^{high}CD9^{high} monocytes subset. *P* value was calculated using two-sided unpaired Student's t-test.

c Gating strategy for Treg cells. Lymphocytes were gated on CD3 and CD4. And CD3^{+}CD4^{+} cells were further gated on CD25 and FOXP3.

Supplementary table titles

Table S1 All cluster marker genes for each clustering and sub-clustering of the scRNA-seq data.

Table S2 Cell number of each donor in each cluster of each clustering and sub-clustering.

Table S3 DEGs of skin immune cells in the scRNA-seq data of the discovery cohort.

Table S4 Functional enrichment analysis using CD8^{+} T cells-specific DEGs of skin scRNA-seq data.

Table S5 Functional enrichment analysis using DEGs of Langerhans cells of skin scRNA-seq data.

Table S6 Ligand and receptor interactions of skin immune cells of the discovery cohort.

Table S7 Ligand and receptor interactions of PBMCs of the discovery cohort.

Table S8 Patients information. For acid-fast staining, two "+" indicated 1 to 10 acid-fast bacteria were observed in every 10 view field of microscopes (1,000 magnification); three "+" indicated 1 to 10 acid-fast bacteria were observed in each view field of microscopes; four "+" indicated 10 to 100 acid-fast bacteria were observed in each view field of microscopes; five "+" indicated 100 to 1,000 acid-fast bacteria were observed

in each view field of microscopes; six “+” indicated more than 1,000 acid-fast bacteria or clump of bacteria were observed in each view field of microscopes.
